# Preparation and Characterization of Niosomes for Bacteriophage Delivery

**DOI:** 10.1002/smsc.202500530

**Published:** 2025-12-19

**Authors:** Ashley Hannah George, Petr Jelinek, Martin Benešík, Simona Košiarčiková, Jiří Mikšátko, Ivona Pečurlić, Marek Moša, Miroslav Šoóš

**Affiliations:** ^1^ Department of Chemical Engineering University of Chemistry and Technology Prague Technická 5 166 28 Prague 6 Czech Republic; ^2^ MB Pharma s.r.o. 120 00 Prague Czech Republic; ^3^ Imaging Methods Core Facility BIOCEV Faculty of Science Charles University 252 50 Vestec Czech Republic

**Keywords:** antibacterial therapy, bacteria, bacteriophages, nanovesicles, niosomes, stearylamine

## Abstract

Vesicular nanocarriers, such as niosomes, are versatile systems for delivering therapeutic agents, including small molecules, proteins, enzymes, nucleic acids, and other biologics. Herein, the encapsulation of bacteriophages within niosomes is investigated, expanding the conventional application of these carriers. Formulations are prepared with varying concentrations of stearylamine, a cationic cosurfactant, to assess the interactions between phages and vesicular membranes. They are characterized by dynamic light scattering, zeta potential analysis, and viral titration, providing insights into vesicle stability and phage encapsulation efficiency. Based on the characterization analysis, an optimal concentration of stearylamine is determined for successful phage encapsulation, as confirmed by cryo‐electron microscopy. The stability and activity of encapsulated phages are further evaluated through pH stability tests and in vitro kinetic assays. These findings demonstrate the potential of niosomes as effective carriers for bacteriophage delivery and highlight their broader applicability for encapsulating other unconventional or sensitive therapeutic agents, offering a promising strategy for antibacterial applications.

## Graphical abstract

Incorporation of stearylamine into the niosome bilayer imparts a positive surface charge that promotes electrostatic interactions with negatively charged bacteriophages. This modification facilitates efficient phage encapsulation while preserving phage activity, demonstrating a simple strategy to enhance niosome‐based delivery systems for phage therapy.

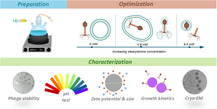

## 1 Introduction

Niosomes are versatile nanocarriers composed of nonionic surfactants and cholesterol,^[^ ^1^ ^]^ with membrane stabilizers or cosurfactants optionally included to enhance the functionality of the formulation.^[^ ^2^, ^3^ ^]^ These soft nanovesicular structures have been extensively researched to encapsulate active pharmaceutical ingredients (APIs) for controlled and targeted delivery.^[^ ^4^ ^]^ Their amphiphilic structure enables them to encapsulate both lipophilic and hydrophilic compounds, making them adaptable for therapeutic and diagnostic applications.^[^ ^5^ ^]^ While niosomal research has been traditionally focused on smaller molecules, such as APIs, proteins, peptides,^[^ ^6^, ^7^ ^]^ and genetic material,^[^ ^8^, ^9^, ^10^ ^]^ their potential to deliver complex and larger biologics has not been fully explored.

Bacteriophages, viruses that selectively infect and eliminate bacteria hosts, represent an alternative class of biotherapeutics capable of addressing the global challenge of multidrug‐resistant (MDR) infections. The virion sizes typically ranging from 20 to 200 nm, depending on the type of isolate.^[^ ^11^ ^]^ The overuse of antibiotics has accelerated the threat of a postantibiotic era due to the emergence and spread of MDR bacteria.^[^ ^12^ ^]^ Currently, the most critical bacterial pathogens are referred to as ESKAPE (*Enterococcus faecium*, *Staphylococcus aureus*, *Klebsiella pneumoniae*, *Acinetobacter baumannii*, *Pseudomonas aeruginosa*, and *Enterobacter* species), which is a group of six resistant strains responsible for nosocomial or hospital‐acquired infections.^[^ ^13^ ^]^ Unlike conventional antibiotics, phages possess an auto dosing capability, increasing their concentration at the site of infection in proportion to the availability of their bacterial host.^[^ ^14^ ^]^ Bacteriophages offer a viable alternative to antibiotics; however, their therapeutic effectiveness is highly dependent on the delivery strategies.^[^ ^15^ ^]^ They are sensitive and susceptible to inactivation by environmental conditions such as pH, temperature, and enzymatic degradation.^[^ ^16^ ^]^ Without suitable formulations, phages may lose infectivity before reaching their target bacteria, limiting their efficacy.

To overcome these limitations, there is a growing interest in developing advanced delivery systems that can stabilize phages, protect them from hostile biological environments, and enhance their therapeutic potential.^[^ ^17^ ^]^ Nanocarrier‐based encapsulation presents a promising strategy to shield phages from external stressors while enabling controlled release and targeted delivery. Among various nanocarrier platforms such as liposomes,^[^ ^18^ ^]^ polymeric nanoparticles,^[^ ^19^ ^]^ and nanoemulsions,^[^ ^20^, ^21^ ^]^ niosomes provide a unique combination of tunability, biocompatibility, and cost‐effective formulation, making them suitable for the delivery of biologics.

In this study, we focus on the development of stearylamine‐modified niosomal vesicles for the encapsulation of bacteriophages as a model system for large biotherapeutic agents. By optimizing vesicle composition and surface charge, we aim to enhance phage delivery. We further evaluate the physicochemical characteristics of the formulations, assess the protection against acidic and basic conditions, and determine the retention of phage infectivity postencapsulation. This work addresses a key gap in phage formulation research by providing insight into how soft vesicular nanocarriers can be tuned to accommodate and protect structurally complex biologics during administration.

## 2 Results and Discussion

### 2.1 Size Analysis of Formulations

The modified injection method successfully produced formulations without any instability, aggregation, or contamination, demonstrating that the setup is effective for preparing niosomes under aseptic conditions. Dynamic light scattering (DLS) at a scattering angle of 90° was employed to determine the hydrodynamic diameter and polydispersity index (PDI) of all the samples as shown in **Table** **1**.

**Table 1:** Native phage, plain niosomes (Nio), and niosome‐phage (Nio‐Ph) samples with their diameters and PDI measured by DLS (mean ± SD; *n* = 3).

Sample	Stearylamine [mM]	Diameter [nm]	PDI
DSM 33473 phage	–	208.5 ± 8.7	0.5 ± 0.1
Nio0	0.0	261.3 ± 3.3	0.2 ± 0.2
Nio1	4.8	286.9 ± 2.3	0.2 ± 0.1
Nio2	19.2	270.4 ± 13.5	0.2 ± 0.1
Nio‐Ph1	0.0	251.4 ± 3.8	0.1 ± 0.1
Nio‐Ph2	0.3	273.7 ± 4.2	0.1 ± 0.1
Nio‐Ph3	0.6	279.7 ± 6.7	0.1 ± 0.1
Nio‐Ph4	0.9	273.6 ± 2.9	0.1 ± 0.1
Nio‐Ph5	1.2	267.1 ± 4.9	0.1 ± 0.1
Nio‐Ph6	2.4	257.0 ± 19.9	0.2 ± 0.1
Nio‐Ph7	4.8	316.7 ± 10.9	0.2 ± 0.1
Nio‐Ph8	6.0	329.8 ± 12.3	0.2 ± 0.1
Nio‐Ph9	9.6	379.1 ± 3.8	0.3 ± 0.1
Nio‐Ph10	19.2	449.3 ± 7.2	0.2 ± 0.1

The native phage sample showed an average hydrodynamic diameter of about 200 nm. In contrast, cryogenic electron microscopy (cyro‐EM) imaging and subsequent image analysis of the lysate revealed an average phage length (from head to end of tail) of 245.3 ± 27.8 nm (see Figure S1, Supporting Information). Although DLS slightly underestimates the true length due to the assumption of spherical particles,^[^ ^22^ ^]^ it remains a reliable tool for monitoring the size of complex formulations, such as phage–niosome systems.

Blank niosomes (Nio0–Nio2) had hydrodynamic diameters of 261–287 nm with low PDI (≈0.2), indicating uniform vesicle formation. Nio1 was slightly larger than Nio0, while Nio2 showed greater variability in size possibly due to the greater amount of stearylamine in the formulation. Incorporation of phages in niosomes led to an increase in diameter at stearylamine concentrations ≥4.8 mM in comparison with blank niosomes, even reaching up to 449.3 nm at 19.2 mM, likely reflecting electrostatic interactions between negatively charged phages and cationic niosomes that promote complex formation or encapsulation. PDI values remained low (0.1–0.3), maintaining monodisperse populations at higher stearylamine concentrations. The modified injection method produced niosomes of 250–450 nm, significantly smaller than the ≈800 nm phage‐loaded vesicles obtained by González‐Menéndez et al.^[^ ^16^ ^]^ using manual shaking and homogenization of a commercial Pronanosome‐Nio‐NTM product (Nanovex Biotechnologies).

### 2.2 Zeta Potential and Phage Viability

**Figure 1:**
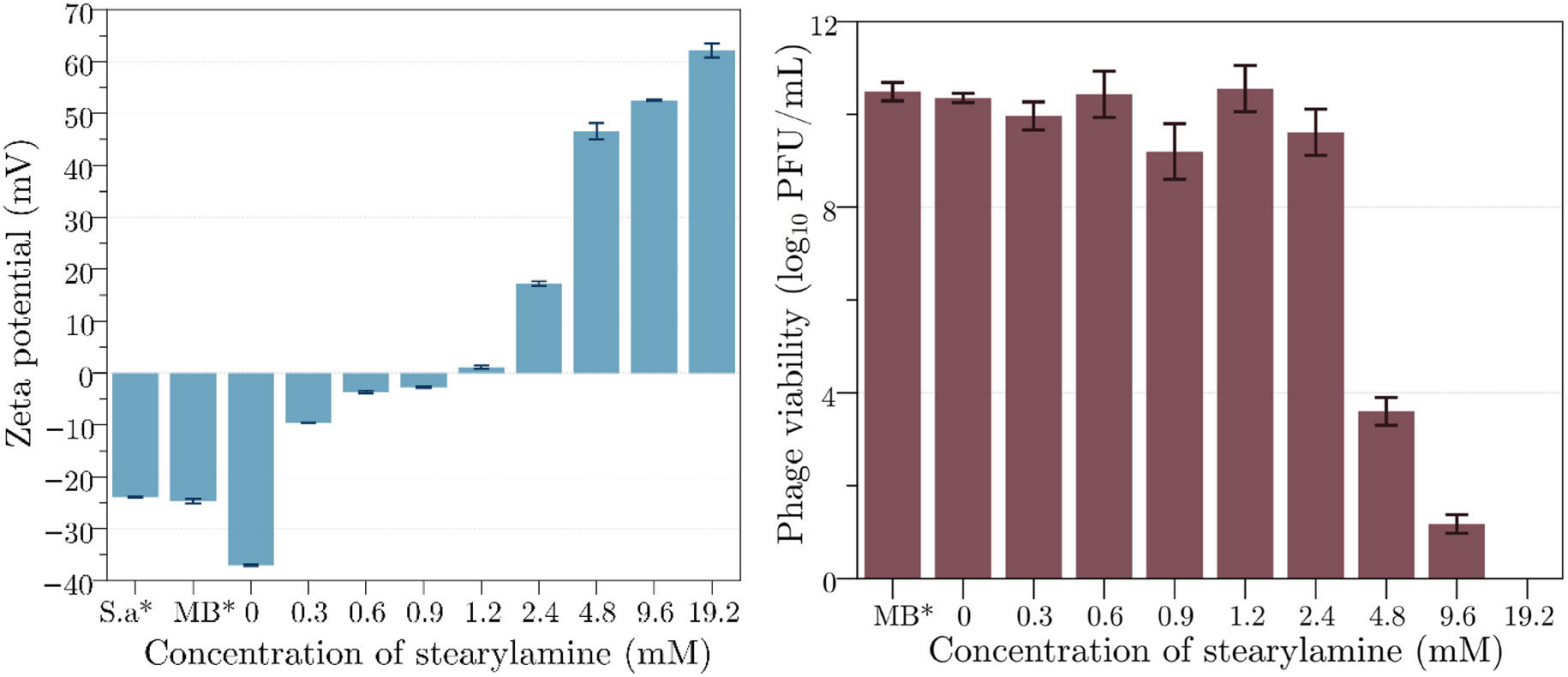
Zeta potentials of plain bacteria *S. aureus* (S.a^*^), phage (MB^*^), and niosome‐phage formulations (left) and the phage viability of each formulation (right). The results are presented as mean ± SD (*n* = 3).

The stability and effectiveness of niosome formulations containing bacteriophages are critically influenced by their surface charge, as indicated by zeta potential and phage viability measurements in **Figure** **1**. In this study, we evaluated the impact of varying concentrations of stearylamine on the zeta potential of niosomes. This cationic cosurfactant enhances the electrostatic properties of the niosomes, which is an important factor for maintaining colloidal stability and preventing aggregation.^[^ ^3^, ^23^ ^]^ These results demonstrate a concentration‐dependent increase in zeta potential, shifting the niosomal surface charge toward extremely positive values (Table S3, Supporting Information shows zeta potential values of Nio1 and Nio2), providing insights into the optimization of niosome formulations for phage delivery. The surface charge of bacteria, phages, and blank niosomes was initially negative, as reflected in their zeta potential values. Incorporation of increasing amounts of cosurfactant stearylamine into niosomal formulations gradually shifted the zeta potential from negative to positive, reaching values above +60 mV at the highest concentration tested (19.2 mM). The effect of stearylamine on phage viability was evaluated in parallel. At low concentrations, phage titers remained comparable to those of the native phage lysate (MB^*^), suggesting that moderate levels of stearylamine allow phage association with niosomes without compromising infectivity. However, as the stearylamine concentration exceeded 4.8 mM, a decline in viable phage counts was observed.

Phage titers drastically decreased by 7 log units at 9.6 mM and at 19.2 mM only 1 log PFU mL^−1^ of phages remained. These findings demonstrate that while stearylamine incorporation enables charge‐driven interactions between phages and niosomes with excessively high positive surface charge destabilizing the phage structure, leading to a loss of viability. To investigate whether the observed phage deactivation was due to the surface charge or the vesiculation process, blank Nio2 (with a high zeta potential of 78.4 mV) was mixed with native phage in equal proportions (see Table S1, Supporting Information). The resulting zeta potentials were comparable to those of the encapsulated phages, indicating that this ratio was appropriate for the study. No inactivation or reduction in phage titer was observed upon mixing and incubating for 1 day, suggesting that the phage deactivation was not charge‐mediated but rather a consequence of the vesiculation process.

To assess the storage stability of the optimized formulation, Nio‐Ph7 was monitored for a month. The diameter, zeta potential, and phage viability remained comparable to the values obtained immediately after preparation (see Table S2, Supporting Information), confirming that the formulation maintains its physicochemical properties and biological activity over this period.

**Figure 2:**
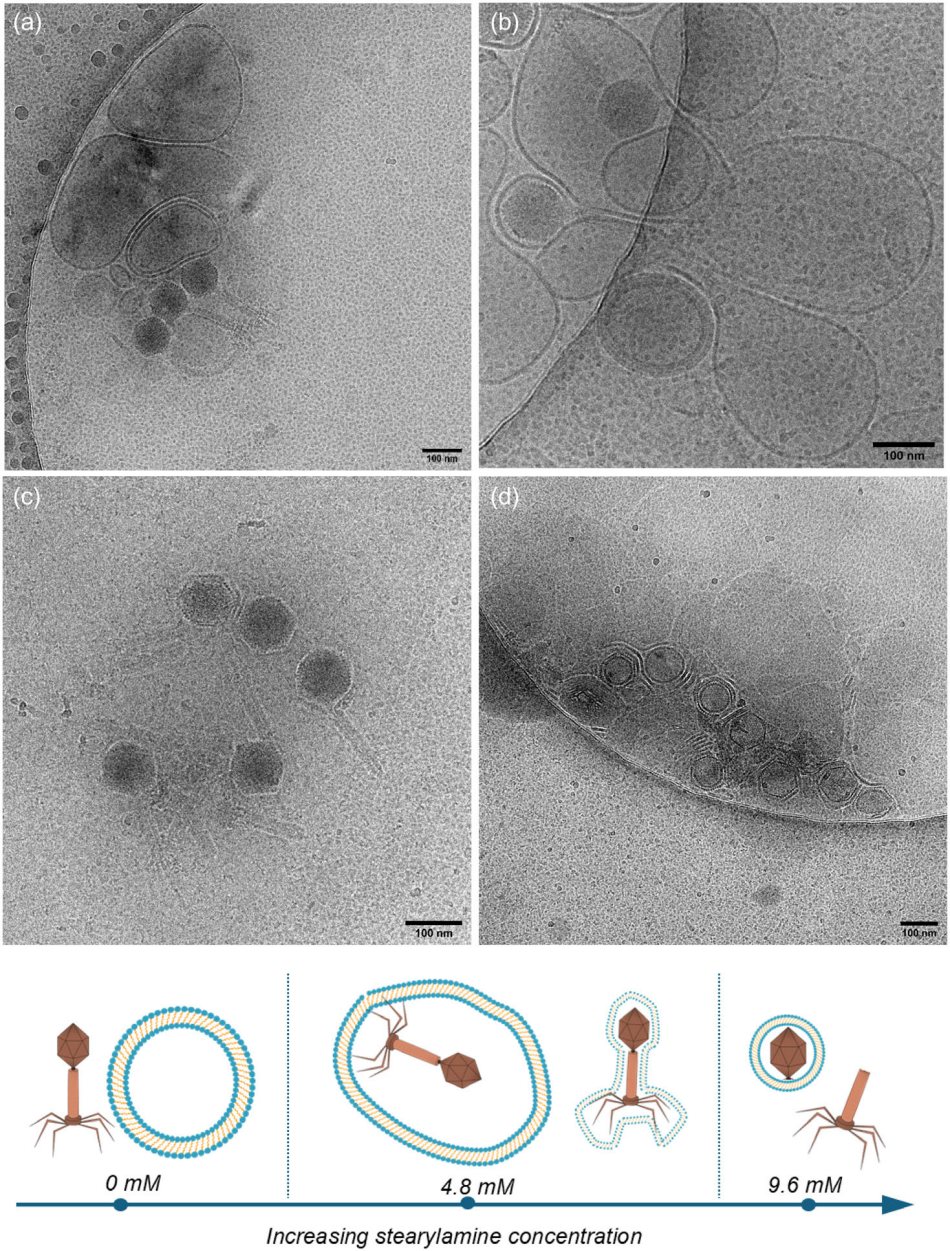
Cryo‐EM images of selected niosomal formulations containing phages: a) 1.2 mM of stearyl amine, b,c) 4.8 mM of stearylamine, and d) 9.6 mM of stearylamine. Scale bar for all images is 100 nm. Illustrative schematic of phage encapsulation as concentration of stearylamine increases (bottom).

Cryo‐EM was performed on selected formulations to investigate the behavior of phages and vesicle formation upon the addition of stearylamine. As shown in **Figure** **2**a, the Nio‐Ph1 formulation displays phages localized near vesicles, indicating minimal direct interaction between the two entities. In contrast, Nio‐Ph7, identified as the optimal formulation based on zeta potential and titer analyses, exhibited two distinct modes of encapsulation (Figure 2b,c): some phages were enclosed within larger vesicles, while others were individually wrapped by a vesicular coat. These observations suggest that electrostatic interactions facilitate encapsulation.

At the highest stearylamine concentration (9.6 mM, Nio‐Ph9), a strong interaction between phages and vesicles was evident, resulting in structural disruption of the phage, notably the detachment of phage tails from their heads (Figure 2d). This concentration‐dependent loss on the phages structure is likely driven by mechanical stress in combination with the high positive charge during the vesiculation process. Consistent with this, Tahara et al.^[^ ^24^ ^]^ reported that cationic stearylamine liposomes inhibit baculovirus infectivity by destabilizing viral membranes through electrostatic interactions. While their study demonstrated inhibition by incubating preformed cationic liposomes with the virus, our results suggest that when phages are present during vesicular self‐assembly, the combination of high charge density and mechanical forces leads to phage destabilization. These observations indicate that increasing stearylamine concentration enhances phage–vesicle interactions, promoting encapsulation at moderate concentrations but causing structural disruption of phages at higher concentrations.

**Figure 3:**
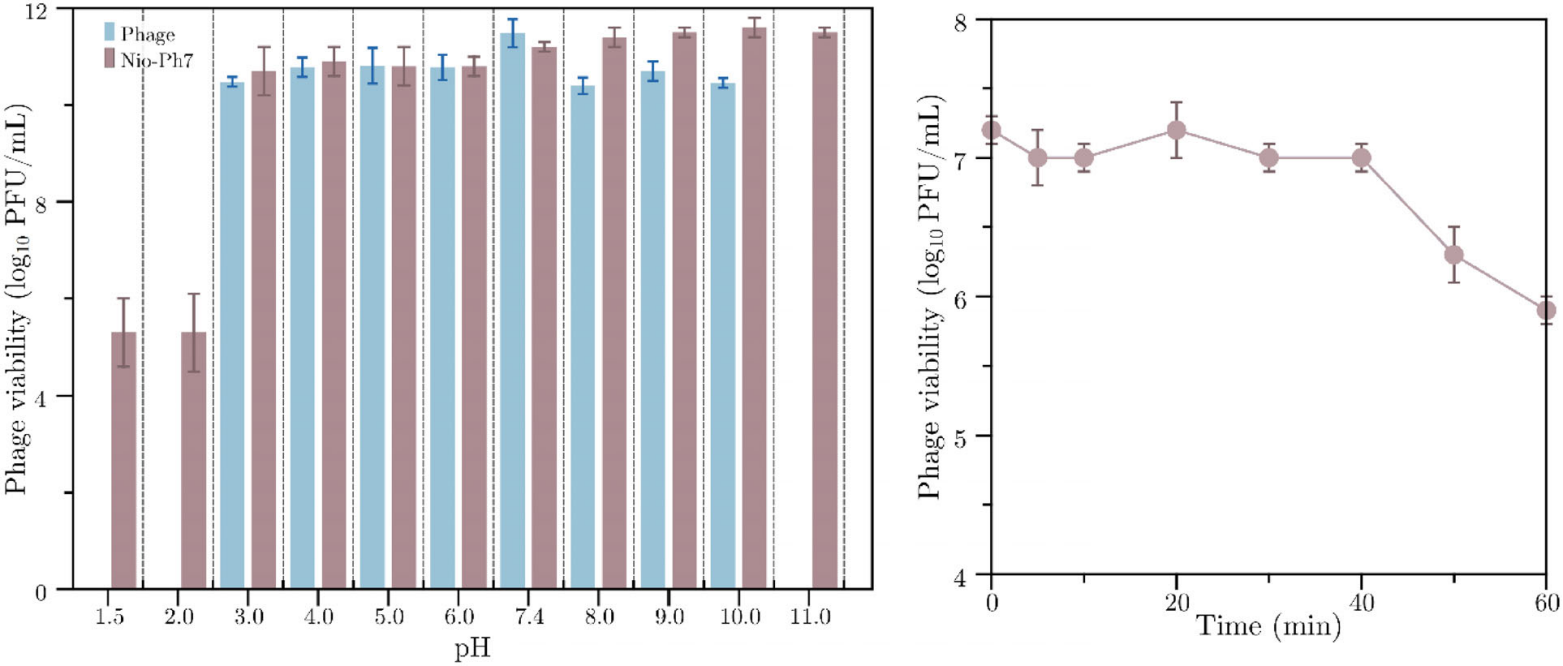
Native phage and niosome‐phage formulation (Nio‐ph7) incubated in pH 1.5–11 buffers (left). Time dependent stability of Nio‐ph7 in pH 1.5 buffer (right). Mean ± SD (*n* = 3).

The stability of phage‐loaded niosomes (Nio‐Ph7) and free phage lysates was evaluated across a broad pH range (1.5–11.0). As shown in **Figure** **3** (left), free phage particles demonstrated viability from pH 3.0 to 10.0, with titers remaining consistently above 10 log PFU mL^−1^. However, exposure to strongly acidic (pH 1.5 and 2.0) and highly alkaline (pH 11.0) conditions resulted in complete loss of phage activity, highlighting the sensitivity of native virions to extreme environments. In contrast, phage encapsulated within the Nio‐Ph7 formulation retained significant stability across a wider pH range (3.0–11.0), and even under extreme acidic conditions (pH 1.5 and 2.0), titers dropped by ≈5 log units indicating substantial protective effects of the niosomal carrier.

To further assess stability under acidic stress, the kinetics of Nio‐Ph7 titer loss at pH 1.5 were monitored over 60 min (Figure 3, right). An immediate reduction in phage viability was observed, with titers dropping from 10 to 7 log PFU mL^−1^ at time zero (titration done immediately after mixing) to 6 log PFU mL^−1^ within the first 20 min. Continued incubation led to a further gradual decline, ultimately reaching ≈6 log PFU mL^−1^ at 60 min. These results show that while extreme acidity induces a notable reduction in infective phage count, niosomal encapsulation still offers meaningful protection compared to unformulated phages. Moreover, the same sample was analyzed after 1 week of incubation in low‐pH media and the titer remained unchanged.

### 2.3 In Vitro Bacterial Growth Assessment

**Figure 4:**
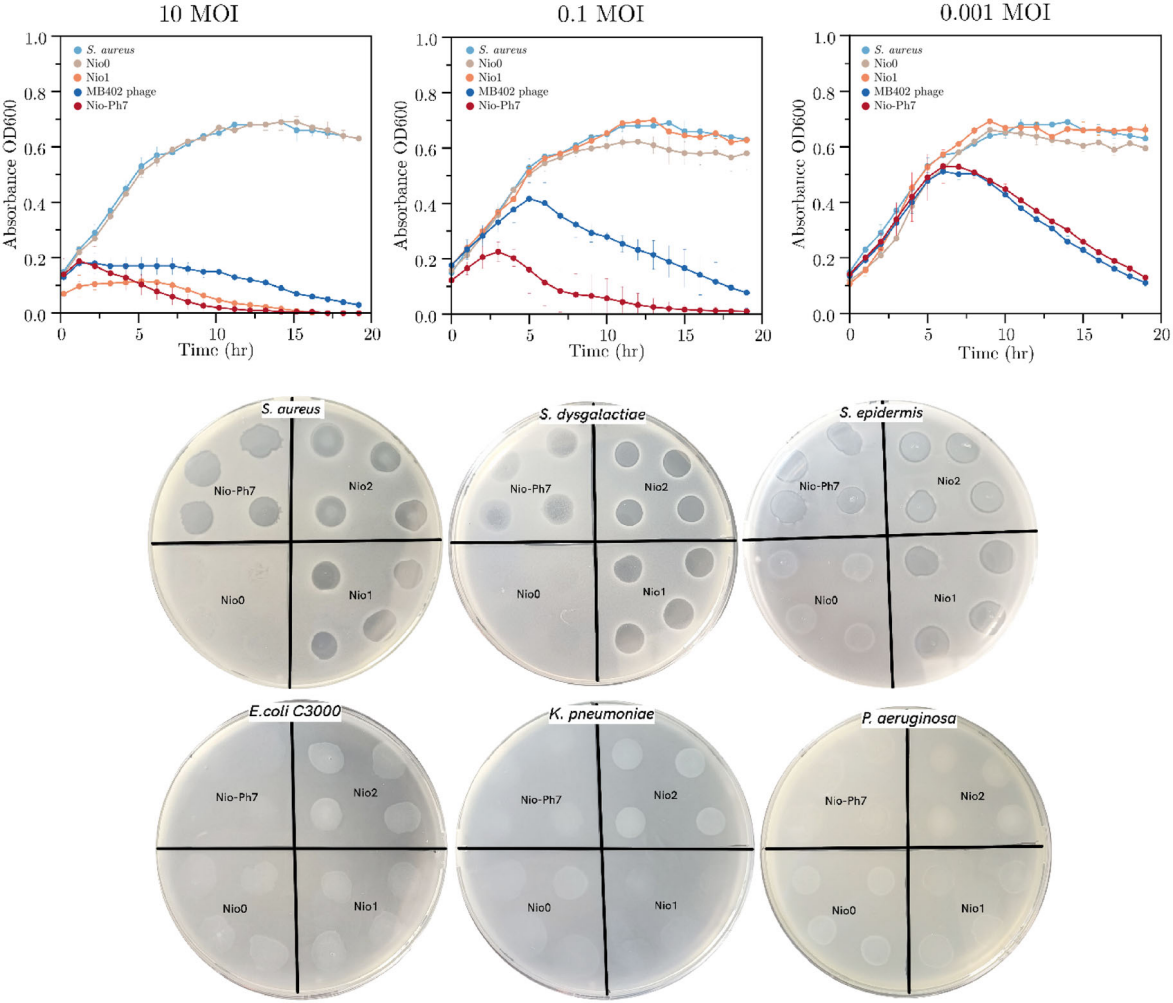
In vitro kinetics of bacteria growth at different MOIs: 10, 0.1, and 0.001 of plain phage, blank niosomes (Nio0 and Nio1), and optimal Nio‐Ph7 formulation (Top row). The *S. aureus* (control) curve represents the growth of bacteria in media. Control of blank niosomes (Nio0‐Nio2) and Nio‐Ph7 against Gram‐positive bacteria: *S. aureus*, *S. dysgalactiae*, and *S. epidermis* and Gram‐negative bacteria: *E. coli C3000*, *K. pneumoniae*, and *P. aeruginosa*.

The growth curves shown in **Figure** **4** represent the bacterial growth in media based on the turbidity of the suspension measured at 600 nm wavelength by UV–vis spectrometer. The control curve containing only *S. aureus* enters the stationary phase approximately after 10 h. When the phage lysate DSM 33473 is included, the growth declines as a result of the bacterial cell lysis by the phage. For 10 MOI, the Nio‐Ph7 has a steeper decay than the plain phage, which can be explained by the plain niosome curve containing stearylamine (Nio1). By comparing the curves of Nio0 and Nio1, it is confirmed that stearylamine exhibits antibacterial properties against *S. aureus*. Hence, dilutions of Nio‐Ph7 were tested to determine the minimum inhibitory concentration of stearylamine (see Table S1, Supporting Information), which was found to be 1.18 μg mL^−1^, whereas the minimum bactericidal concentration was 118 μg mL^−1^. Bibel et al. also reported antimicrobial activity of stearylamine and sphingosines, achieving a 4‐log reduction at 6.25 μg mL^−1^.^[^ ^25^ ^]^

To avoid the influence of stearylamine in the experiment, a dilution of niosome was selected such that a concentration of stearylamine (0.118 μg mL^−1^) would not affect the bacterial growth, corresponding to the 0.1 MOI graph in Figure 4. Blank niosomes, with and without stearylamine, did not inhibit the bacterial growth and followed the same trend as the *S. aureus* control. DSM 33473 and Nio‐Ph7 showed a decline in growth once again; however, the decline of the latter is more prominent and reaches complete inhibition rapidly. Previous studies on niosome‐mediated antibiotic delivery^[^ ^26^ ^]^ have shown that either fusion with the bacterial membrane or close contact with the bacterial surface can enhance antimicrobial efficacy. By analogy, niosomes may similarly improve the direct interaction of bacteriophages with bacterial cells while protecting them from degradation during delivery, thereby enhancing phage antibacterial activity. At an MOI of 0.001, the growth curves of both the native phage and Nio‐Ph7 overlap, suggesting that niosomes exert minimal effect under such extreme dilution. Bacterial growth is pronounced during the first 5 h, nearly reaching the growth plateau (corresponding to an OD600 of ≈0.65). Beyond this point, phage activity is evident, resulting in a gradual and sustained decline in bacterial growth.

Stearylamine was evaluated against multiple bacterial strains to assess its antibacterial activity. Due to the insoluble nature of stearylamine, blank niosomes were employed as a control to determine any inhibitory effects on the various Gram‐positive and Gram‐negative strains. The results demonstrated that stearylamine exhibited antibacterial activity against all tested Gram‐positive bacteria, including *S. aureus*, *S. dysgalactiae*, and *S. epidermidis*. In contrast, no antibacterial activity was observed against Gram‐negative bacteria. This selective activity can be attributed to the differences in the bacterial cell wall of the gram types. Gram‐negative bacteria have an outer lipopolysaccharide membrane and inner phospholipid membrane, which together act as a protective barrier,^[^ ^27^ ^]^ preventing stearylamine from accessing and inactivating the bacteria. On the contrary, Gram‐positive bacteria lack the outer membrane but consist of a peptidoglycan layer containing negatively charged teichoic acid, which most likely interacts with stearylamine, disrupting the membrane and leading to cell death. This is consistent with the clear inhibition zones observed for Gram‐positive bacteria during the control experiments.

## 3 Conclusion

Niosomes were successfully formulated to encapsulate bacteriophages via electrostatic interactions with stearylamine. High concentrations of stearylamine (>9.6 mM) destabilized phages, whereas an optimal concentration of 4.8 mM enabled effective encapsulation without loss of phage titer. Encapsulation within niosomes enhanced phage stability under extreme pH conditions, providing full protection at pH 11 and partial protection at acidic pH (1.5 and 2.0), compared to complete inactivation of free phages. In vitro kinetics demonstrated that niosome‐encapsulated phages exhibited enhanced bacterial inactivation compared to free phages, independent of the antibacterial effect of stearylamine. These findings indicate that niosomal encapsulation improves both the stability and antibacterial efficacy of phages, highlighting its potential as a promising strategy for targeting drug‐resistant bacterial infections.

## 4 Experimental Section

4.1

4.1.1

##### Materials

The following chemicals were bought from Sigma–Aldrich (Germany): Span 20, Tween 20, stearylamine, and cholesterol (92.5%), Trizma Base, and Calcium Chloride. Agar Bacteriological (Agar No. 1), Tryptone, Yeast Extract were purchased from Oxoid, Thermo Fisher (United Kingdom). *Staphylococcus* bacteriophage DSM 33473 (genus *Kayvirus*) and bacterial strain *S. aureus* DSM 33466 were provided by MBPharma (Czech Republic). Phage propagation and purification were prepared according to previously published methods.^[^ ^28^, ^29^ ^]^ Ethanol (p.a., 99.5%) was purchased from Penta (Czech Republic).

##### Preparation of Bacteriophage‐Loaded Niosomes

Niosomes were prepared by modifying the standard organic phase injection method^[^ ^30^, ^31^ ^]^ to accommodate the aseptic handling of phages. Unlike the standard method, which is performed at atmospheric pressure and elevated temperatures (i.e., at the boiling point of the solvent), the present technique focuses on vacuum‐assisted solvent removal at a phage‐compatible temperature (37 °C) within a closed sterile system using an inline sterile filter. The vacuum method not only preserves the phage viability but also allows rapid solvent removal, considerably reducing the total preparation time compared to the standard method. All niosomal formulations were prepared using the modified organic phase injection method with fixed volumes of organic and aqueous phases and constant amounts of Span 20, Tween 20, and cholesterol. The niosomal system using 3:1 molar ratio of surfactant to cholesterol was employed as a template for the bacteriophage encapsulation based on our previous work.^[^ ^32^ ^]^ For optimizing the encapsulation process, stearylamine was incorporated as a cosurfactant as it imparts a positive charge on the niosomal membrane,^[^ ^33^ ^]^ making it suitable for encapsulating bacteriophages, which are negatively charged due to the genetic material.^[^ ^34^, ^35^, ^36^ ^]^ The goal was to promote encapsulation through electrostatic interactions between the positively charged membrane components and the negatively charged phages. In terms of safety, Tahara et al. reported that pure stearylamine solution was highly toxic to A549 cells after four hours of incubation; however, stearylamine‐based liposomes showed no decrease in cell viability.^[^ ^24^ ^]^ Similarly, Hemmati et al. evaluated the cytotoxicity of blank niosomes containing stearylamine and found no cytotoxic effects.^[^ ^37^ ^]^

In the literature, the use of Span and Tween surfactant mixtures^[^ ^38^, ^39^, ^40^ ^]^ was reported, where both surfactants were dissolved in the organic phase. Our approach for phage‐loaded niosomes involves dissolving the surfactant in different phases based on their hydrophilic–lipophilic balance. The lipophilic surfactant Span 20 was dissolved in the organic phase, whereas the hydrophilic surfactant Tween 20 was included in the aqueous phase along with the phase lysate. Using surfactants of the same alkyl chain length ensures optimal bilayer formation and stability due to the hydrophobic tail interactions of the surfactants.

**Figure 5:**
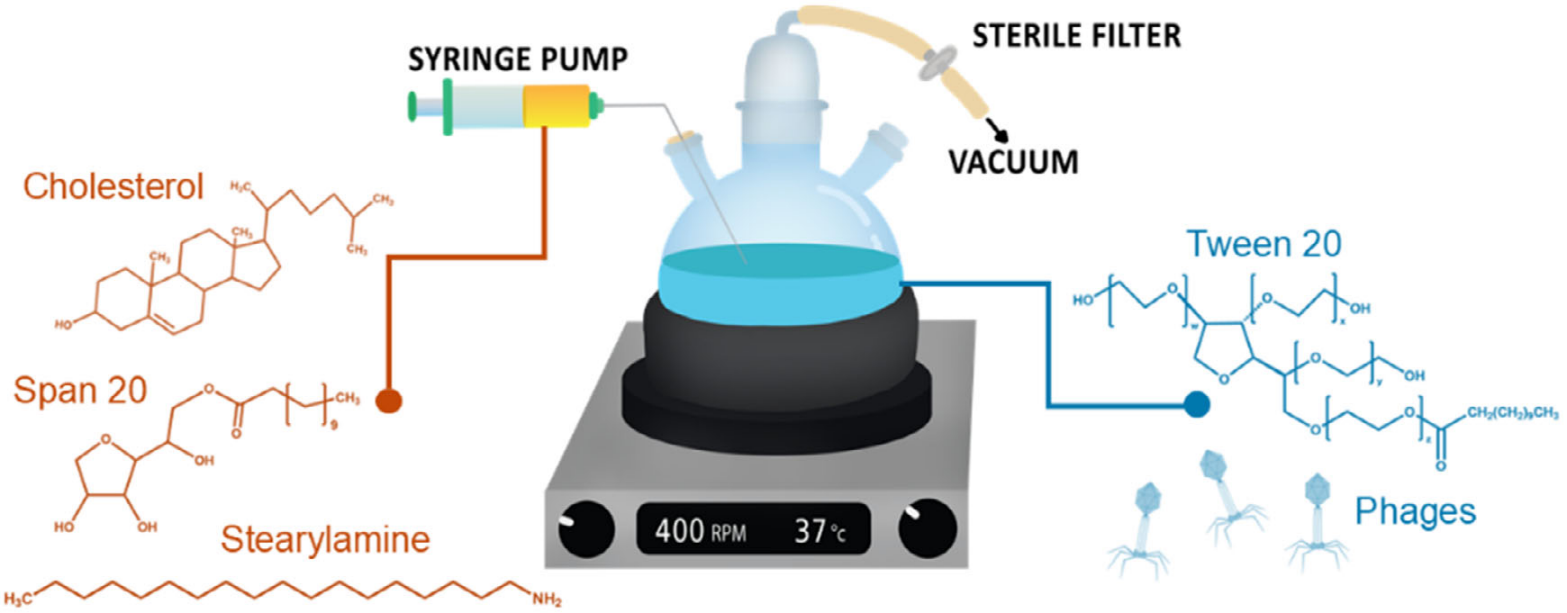
Schematic of experimental setup of niosomal preparation for phage loading.

The organic phase consisted of bilayer components, specifically Span 20 (50 mg), stearylamine (varying amounts), and cholesterol (18.6 mg), dissolved in ethanol (1 mL). The aqueous phase comprised the phage lysate (5 mL) combined with Tween 20 (50 mg). Moreover, sterile conditions were maintained for all components, with those dissolved in ethanol and Tween 20 sterilized by UV exposure prior to use, ensuring that phages would be free from contamination. The entire setup, as shown in **Figure** **5**, was conducted under vacuum and maintained at 37 °C using a three‐neck round‐bottom flask. The top neck was connected via tubing to a sterile filter and then to a gas washing bottle connected to the vacuum pump, and another held a silicone stopper for the injection needle. The organic phase was injected into the aqueous phase at a controlled rate of 0.2 mL min^−1^ with magnetic stirring at 400 rpm. During injection, the vacuum was adjusted so that visible boiling of ethanol occurred, ensuring continuous evaporation of solvent. After the injection was completed, the system was kept under vacuum for an additional 10 min to reduce the residual ethanol content in the final suspension, improving the stability and purity of the formulation.

##### Viral Titration Method

The droplet titration method was employed to determine the concentration of viable bacteriophages in the sample. This method involves preparing a fresh culture of the *S. aureus* DSM 33466 in growth media (7 g L^−1^ tryptone, 10 g L^−1^ yeast extract and 3 g L^−1^ NaCl) and overlaying it with a soft agar mix containing serially diluted phage suspensions. After solidification, 10 μL droplets of each diluted phage suspension (eight dilutions) are applied to the bacterial lawn, allowing phages to infect the bacteria and form clear plaques. Following a 12 h incubation at 37 °C, the number of plaques is counted, and the phage titer is calculated as plaque‐forming units (PFU) per milliliter.

##### Size and Zeta Potential Measurement

The size and zeta potential of the niosome formulations were determined using the dynamic light scattering LS instruments (LS instruments, Switzerland) and Zetasizer Nano‐ZS (Malvern Panalytical). Prior to analysis, the samples were diluted with distilled water to achieve an appropriate concentration for measurement. DLS was utilized to assess the hydrodynamic diameter of the niosomes by measuring the intensity fluctuations of scattered light from a laser source (632.8 nm) directed at the sample. The measurements were conducted at a fixed angle of 90° to ensure optimal detection of the scattered light.

##### Cryo‐EM

Cryo‐EM analysis of phage‐niosomal formulations were performed on samples diluted tenfold and prepared by plunge freezing. 4 μL of sample was applied to glow‐discharged Quantifoil cryo‐EM grids (R 2/1, 300 mesh), blotted for 3 s, and directly vitrified in liquid ethane at −180 °C using a Leica GP2 Plunge Freezer. Grids were transferred under cryogenic conditions to a Simple Origin dual‐grid cryo‐holder and imaged on a Jeol JEM 2100Plus TEM (200 kV) equipped with a LaB_6_ electron gun and TVIPS XF416 CMOS camera. The data were collected using a semiautomated mode with SerialEM software.^[^ ^41^ ^]^ ImageJ software was used for denoising and contrast adjustments.^[^ ^42^ ^]^

##### pH Stability Test

To evaluate the pH stability of phages, 50 mM Tris buffers were prepared and adjusted to a range of pH values (1.5, 2.5, 3.0, 4.0, 5.0, 7.4, 8.0, 9.0, 10, and 11.0) using either hydrochloric acid (HCl) or sodium hydroxide (NaOH). For each pH condition, 100 μL of phage sample was mixed with 900 μL of the corresponding pH‐adjusted Tris buffer. The mixtures were incubated with shaking at room temperature for 1 h. Following incubation, viral titration was conducted in growth mediabiel to determine the viability of phages in both native and encapsulated forms.

##### In Vitro Assessment of Bacterial Inhibition by Phages

Bacterial growth was quantified by optical density measurements at 600 nm (OD_600_) using a UV–vis spectrophotometer (Cary 60, Agilent Technologies, USA). Overnight culture of *S. aureus* was prepared in growth media and incubated at 37 °C.

For bacterial growth monitoring without phage, 1 mL of growth media was transferred into a disposable cuvette, and 200 μL of the overnight bacterial culture was added. The absorbance at 600 nm was recorded immediately (time 0 h) and subsequently at 1 h intervals for the duration of 19 h. For phage–bacteria growth kinetics, 1 mL of growth media was placed into a cuvette, and 10 μL of the diluted treatment sample (either free phage, niosome‐encapsulated phage, or plain niosomes) was added. The spectrophotometer was zeroed against this mixture to eliminate background absorbance. Subsequently, 200 μL of the overnight bacterial culture was added to the cuvette, and OD_600_ readings were taken every hour for 19 h. All assays were performed in triplicate.

## Supporting Information

Supporting Information is available from the Wiley Online Library or from the author.

## Conflict of Interest

The authors declare no conflict of interest.

## Supporting information

Supplementary Material

## Data Availability

The data that support the findings of this study are available from the corresponding author upon reasonable request.
