# Supplementary material for: Preparation and Characterization of Niosomes for Bacteriophage Delivery
Source: Small Sci. 2025 Dec 19;6(1):e202500530. doi: 10.1002/smsc.202500530 (PMC12854246; doi:10.1002/smsc.202500530)
Supplement: Supplementary file 1 — Supplementary Material [file SMSC-6-e202500530-s001.pdf]

Supplementary Information

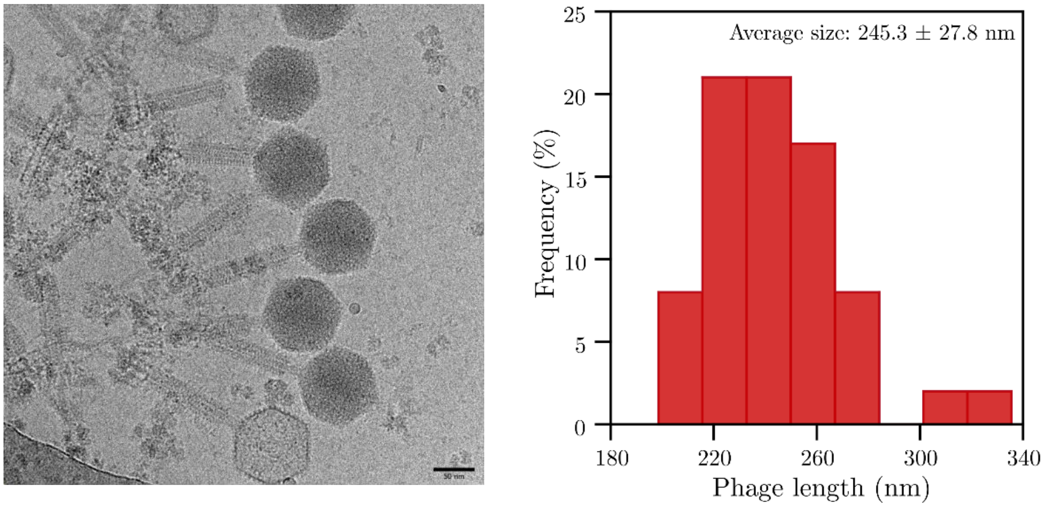

Figure S1 Cryo-EM image of DSM 33473 phage and histogram of phage length measured by image analysis.

Table S1 Characterization of mixture of blank niosomes and phages in equal parts.

	**Nio1**	**Nio2**	**Nio1 + DSM 33473**	**Nio2 + DSM 33473**
DLS	286.9 ± 2.3	270.4 ± 13.5	298.3 ± 5.0	286.1 ± 3.5
Zeta potential	54.0 ± 1.5	78.4 ± 2.9	41.2 ± 1.5	61.5 ± 1.5
Zeta deviation	6.3 ± 0.7	7.5 ± 0.9	5.1 ± 0.6	6.3 ± 0.5
Titer ($\log_{10}$ PFU/mL)	-	-	10.3 ± 0.1	10.0 ± 0.0

Table S2 One month stability of Nio-Ph7 formulation testing DLS, zeta potential, and phage titer.

**Sample**	**Diameter**	**PDI**	**Zeta potential (mV)**	**Titer ($\log_{10}$)**
Day 0	316.7 ± 10.9	0.2 ± 0.1	+46.6 ± 1.6	9.6 ± 0.3
Month 1	336.2 ± 15.6	0.2 ± 0.1	+46.3 ± 5.0	9.4 ± 0.2

Table S3 Concentration of stearylamine in blank niosomes and the OD600 absorbance value after incubation in bacteria culture.

**Concentration of stearylamine (ug/mL)**	**OD_{600} Absorbance**
118	0
58.8	0.21
29.4	0.22
2.35	0.23
1.18	0.36
0.118	0.66
0.0118	0.67
0.00118	0.66
0.000118	0.66
0 (blank)	0.67
